# Psychological Section of the Medico-Legal Society

**Published:** 1893-03

**Authors:** 


**Psychological Section of the Medico-Legal Society.**—This section has been formed under the auspices of the Medico-Legal Society, for the investigation of all branches of Psychological Science. Its affairs will be managed by a committee composed of its executive officers, who also act upon the election of members of the section. These, if members of the Medico-Legal Society, pay as annual dues $1.50 each, and all others $5 each, in advance. The section is interested in all which pertains to the wide domain of psychology; in the rapidly growing facilities which the colleges and universities are offering to students in Experimental work; as well as in that vast region of psychological phenomena, which, with its perplexing and increasing complications, demands the strictest and most scientific investigation. Committees will be appointed from the members of the section for especial study in the departments of Animal Magnetism, Hypnotism, Telepathy and Clairvoyance, and also of the so-called Apparitions, and other claims of respectable Modern Spiritualism. It proposes to conduct these inquiries and investigations with candor] and fairness, upon strictly scientific lines, and to reach, in so far as possible, a valuable and enlightening collection of facts incident to these phenomena, from which important deductions may be made. Applications for membership must be endorsed by some one known to, or properly vouched for by some member of the committee or section. Every student of psychology is invited to unite and co-operate in the labors of this section.

